# Retention of CAD-CAM locator retentive attachment insert in mandibular implant overdenture

**DOI:** 10.1186/s12903-025-05434-3

**Published:** 2025-01-17

**Authors:** Mai Hassan Diab, Medhat Sameh Abdelaziz, Mohamed Hatem Kamal Eldin

**Affiliations:** 1 Removable Prosthodontics Department, Faculty of Dentistry, October 6 University, Giza, Egypt; 2 Faculty of Oral and Dental Medicine, Prosthodontics Department, Future University in Egypt, Cairo, Egypt

**Keywords:** Computer, Aided design, Denture precision attachment, Dental implantation, Denture retention

## Abstract

**Background:**

The continuous development in digital prosthodontics allowed the customization of attachments and retentive inserts which offers an easy and cheap solution for regular maintenance of locator overdentures during daily practice. The present study compared the change in retention values of the fully digitally manufactured custom-made locator attachment retentive insert with the ready-made ones after insertion, removal, and masticatory cycles.

**Methods:**

A complete denture was constructed over a mandibular edentulous epoxy model. Two implants were inserted into the model between the laterals and canines following the prosthetically driven implant protocol. Locator retentive attachment inserts were digitally designed using free-form modeling software and milled from PEEK (Poly Ether Ether Ketone). After the pick-up of the ready- and custom-made retentive inserts, an insertion, removal, and masticatory cycles test simulating 1 year of patient usage was performed. The change in retention values was recorded at baseline, 6 months, and after one year of simulated clinical use. An independent sample t-test was used to compare the data between the two studied groups.

**Results:**

There were statistically significant differences in retention values between the custom-made and ready-made locator inserts at baseline and after 6 months. (*p* = 0.001*). On the other hand, there was no significant difference in retention after 1 year of simulated use. (*p* = 0.083, NS)

**Conclusions:**

The custom-made milled locator retentive attachment insert can be used as an alternative to the ready-made one due to their comparable retention values after 1 year of simulated use.

## Introduction

Implants retained and/or supported mandibular overdentures have greatly influenced patients’ quality of life compared to tissue-supported conventional complete dentures [1–3]. 

The main use of dental attachments is to provide retention for implants or tooth-retained overdentures, Attachment selection is influenced by many factors, such as inter occlusal space, implant angulation and numbers, ease of maintenance, and prostheses type [4]. Locator attachment is the most popular dental attachment as it corrects implant angulation up to 20 degrees with ease of overdenture insertion and removal. It can also be used in limiter inter-arch space with the ability to offer double retention from its outer and inner surfaces [5]. 

The locator attachment consists of a metal abutment or patrix screwed to the implant fixture and a changeable matrix or retentive insert inserted in the denture fitting surface [6]. The locator retentive insert can be fabricated from Poly Oxy Methylene (POM), Poly Ether Ether Ketone (PEEK), or Polyvinylsiloxane (PVS) [7–10]. 

The retentive inserts undergo wear and inner surface deterioration due to long-term masticatory cycles and daily insertion and removal by the patient [7]. Patient satisfaction with the locator overdenture is mainly influenced by the retention of the prosthesis, and one-third of overdenture complications are related to retention loss, which necessitates regular maintenance visits and regular retentive insert change [11, 12]. 

The continuous development in digital prosthodontics has unleashed the power to design and fabricate many implant-prosthetic components, such as locator retentive inserts [13, 14]. , but the reports regarding the construction of custom-made locator retentive inserts and their validation in comparison to the ready-made ones are lacking.

The custom-made attachments offer a time and cost-effective solution for regular maintenance of implant overdentures therefore, the objective of this comparative present study was to evaluate the digitally fabricated custom-made locator retentive inserts and compare them with the ready-made inserts in terms of retention loss after 1 year of simulated clinical use of insertion, removal, and chewing cycles. The null hypothesis is that either the ready-made or custom-made locator retentive inserts have similar behavior in retention and retention loss in implant retained overdenture.

## Materials and methods

### Fabrication of the study model

An epoxy resin completely edentulous mandibular model (Swiss Chem; construction chemicals, Egypt) was constructed by duplicating an edentulous mandibular stone cast using laboratory silicone material (REPLISIL 22 N, dent-e-con, Germany) [15].

A Cone Beam Computed Tomography (CBCT) scan was performed for the model with the following parameters (80 KVP, 8 MA) to obtain a digital imaging and communication in medicine (DICOM) file, which is later 3D segmented into a standard tessellation language (STL) file. Then An implant drilling guide was constructed for implant placement according to prosthetically driven implant protocol after a virtual setting of teeth using implant planning software (real guide; 3diemme, Italy) [5, 16]. 

**Fig. 1:**
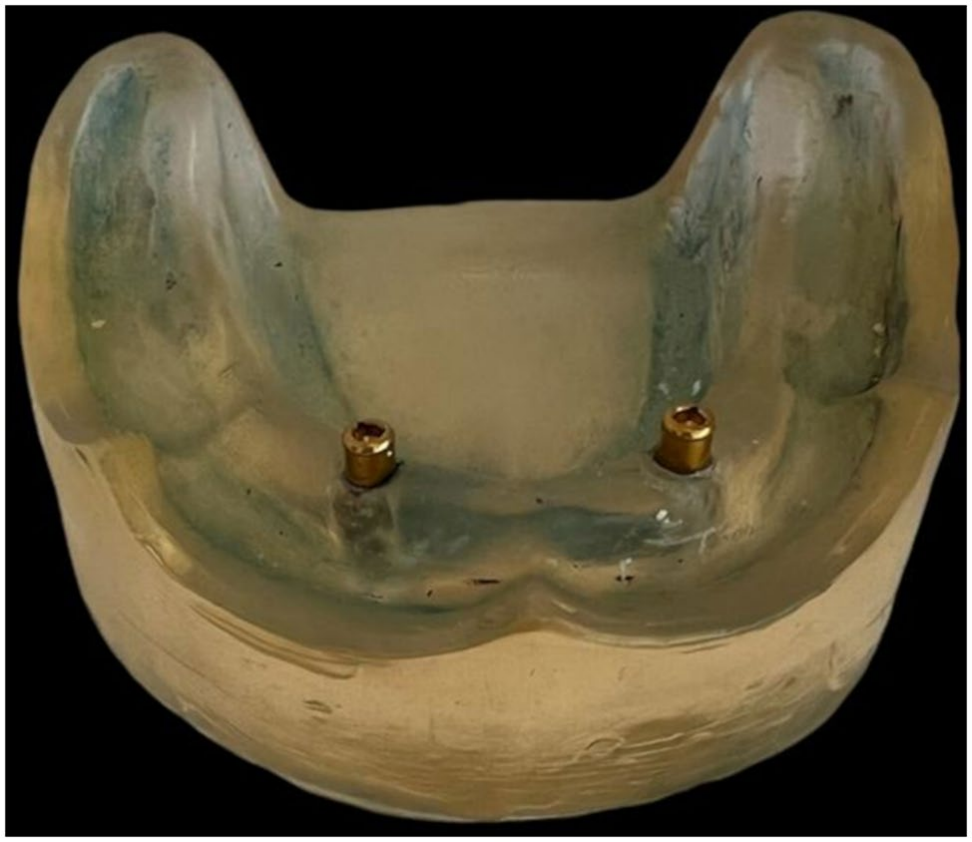
The locator abutments tightened over the implants on the epoxy model

Using a computer-designed implant placement surgical stent, two dental implants 4 mm in diameter and 10 mm in length (SuperLine; Dentium.) were placed bilaterally between the lateral incisor and canine at 22 mm distance apart at zero-degree angle with the two implants perpendicular to the model. Finally, two locator attachments with a 2 mm height, (Dentium, USA) were tightened over the implants with a torque of 25 Ncm as recommended by the manufacturer. (Fig. 1).

### Fabrication of customized digitally milled PEEK cap for Locator attachment

An optical scan was performed for the model with the locator attachments tightened to it using an extraoral optical scanner (Medit T500). Later, the standard tessellation language (STL) file of the model was imported into a free-form modeling software program (MESHMIXER 3.5 software, Autodesk).

**Fig. 2:**
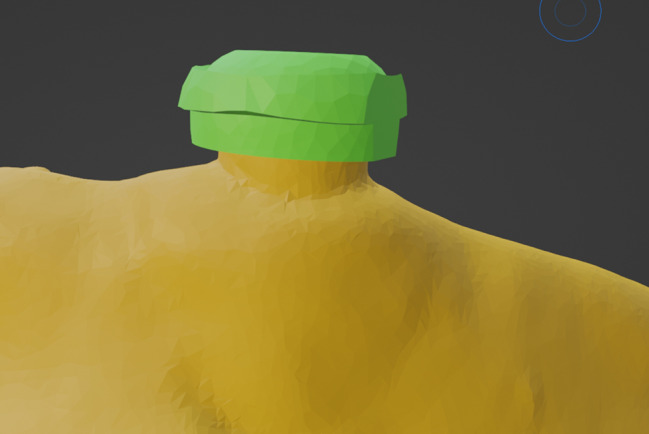
The finalized design of the outer surface of the custom-made locator retentive insert with external undercuts to provide mechanical interlocking with the denture fitting surface

The outline of the custom locator retentive insert was drawn using the software select tool, the retentive insert was given a thickness of 1.5 mm using the extrude software tool, and an offset spacer of 0.03 mm was considered when designing the retentive insert to allow for attachment mobility. Last but not least, and most importantly, circular undercuts were designed on the outer surface of the retentive insert using the attract software tool that will be used later in the pickup step for mechanical interlocking between the denture and the retentive insert [14]. (Fig. 2)

**Fig. 3:**
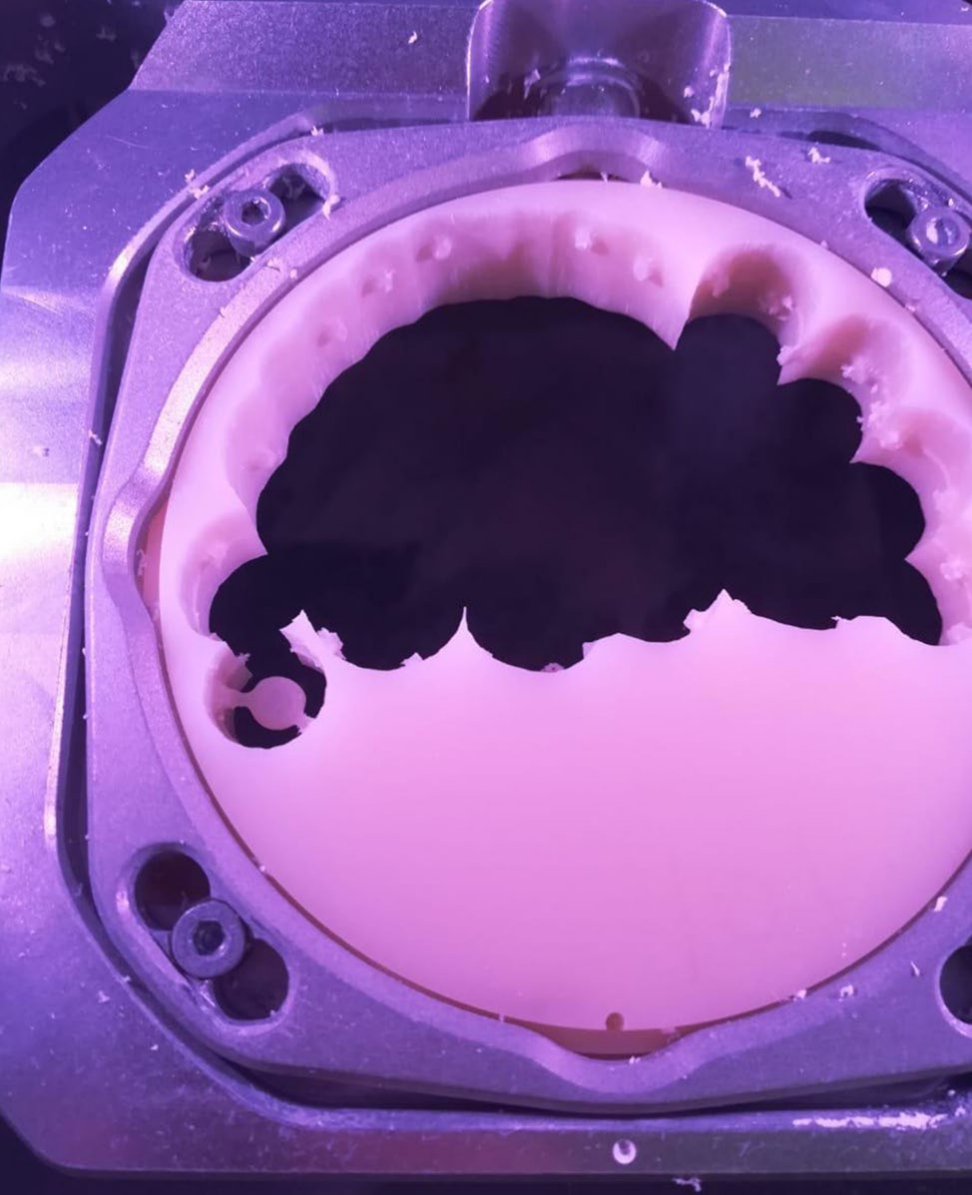
The milled locator retentive insert from the PEEK desk

The final design of the retentive insert was exported in the form of an STL file and milled from a PEEK block using a milling machine (ARUM 5X-450; ARUM Dentistry). (Fig. 3)

### Grouping

**Fig. 4:**
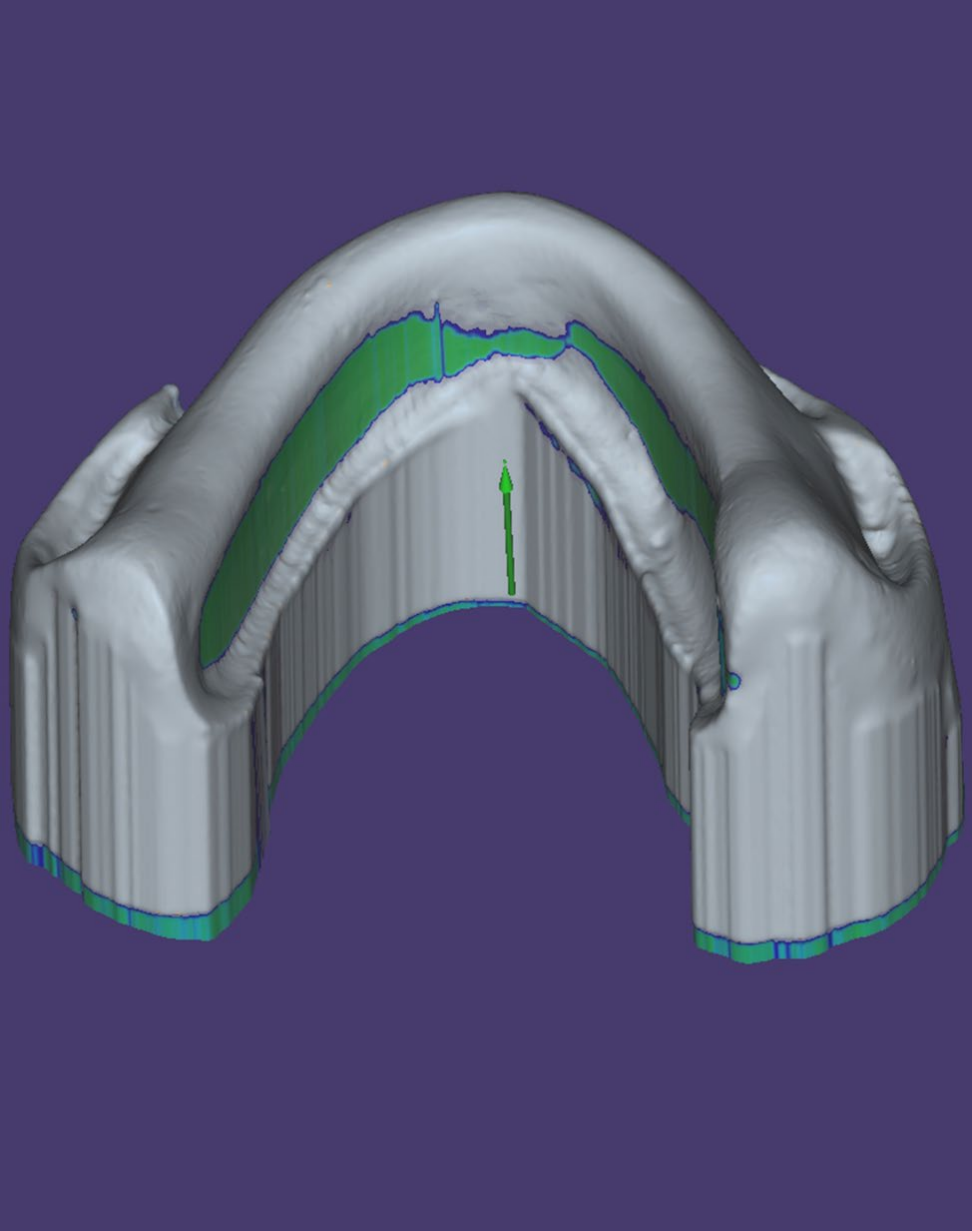
The virtual blocked-out model with a vertical path of insertion

**Fig. 5:**
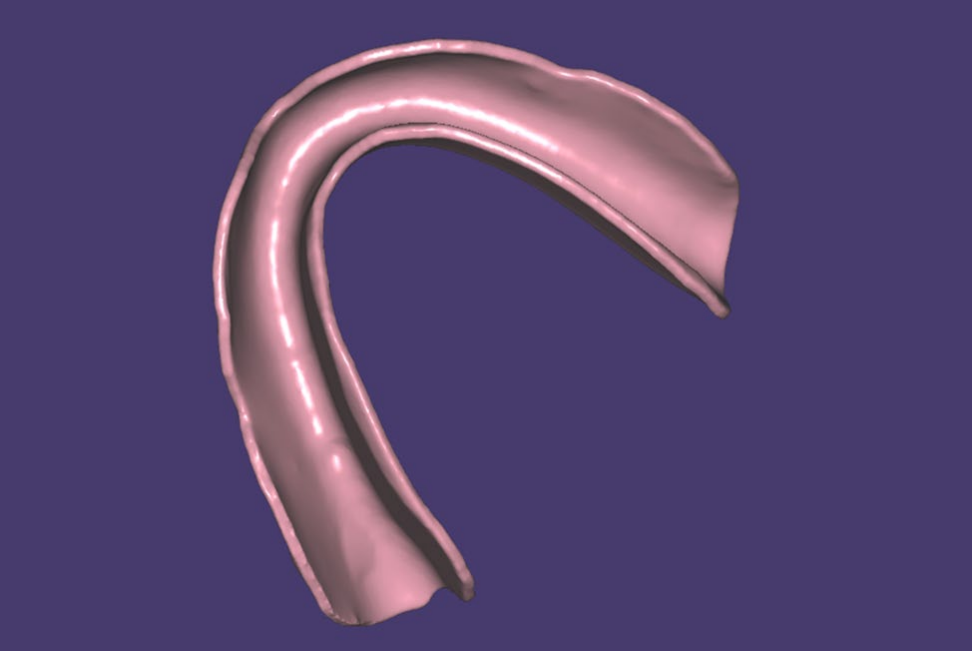
The outline of the virtually designed trial denture base

**Fig. 6:**
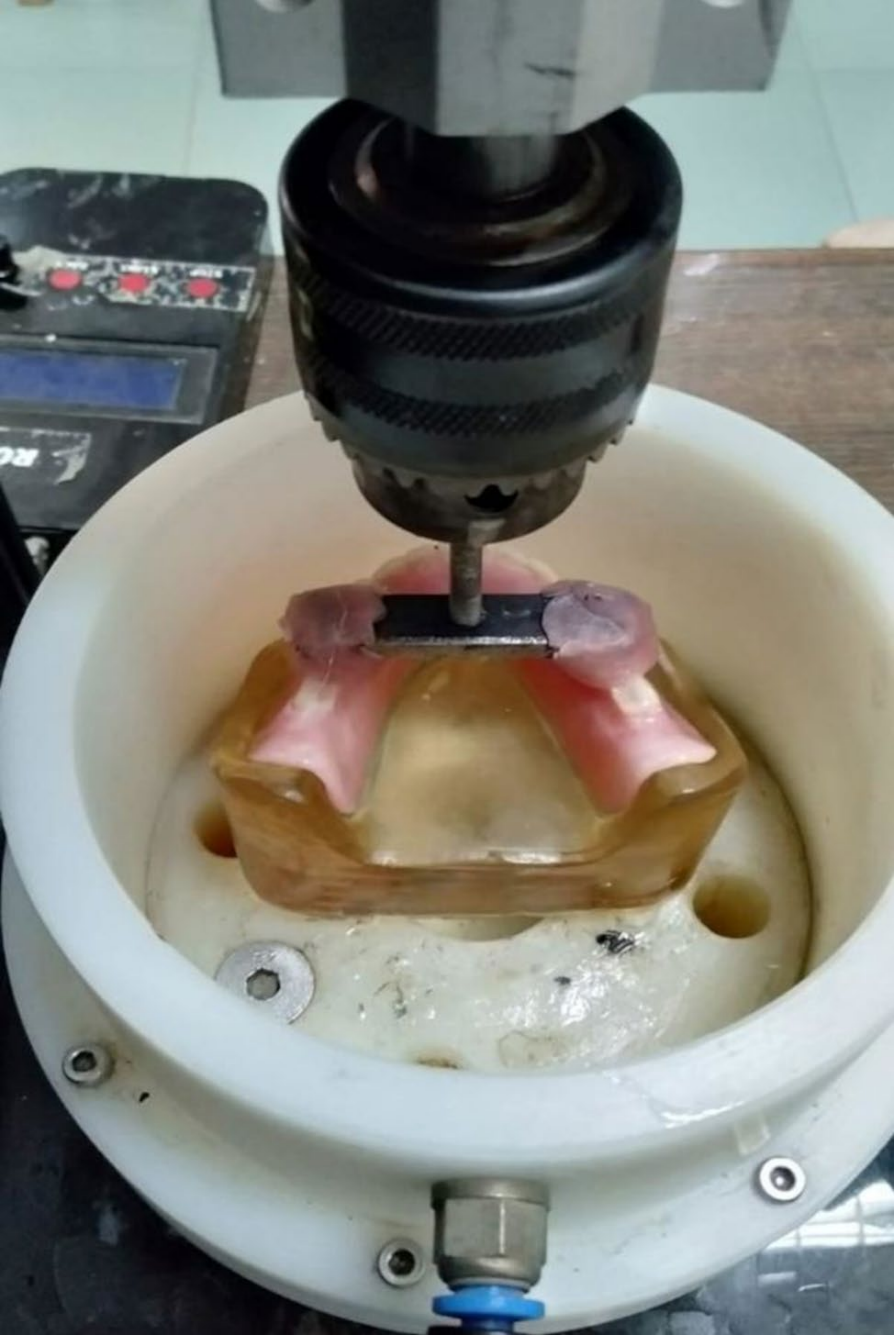
A horizontal rod attached to the denture cameo surface to be used during the chewing simulator and insertion and removal test

Virtual blocking of the undercut was performed after selecting a vertical path of insertion using a computer designing software program (Dental CAD; Exocad GmbH). (Fig. 4) The trial denture base was virtually designed on the block-out model. (Fig. 5) Twenty copies of mandibular trial denture bases were 3D printed in castable resin and then packed with heat-cured resin (Denture Base Material; Acrostone) using metal flasks. A rod was attached to each denture geometric center, which will be later used during measuring the retention values. (Fig. 6)

The study was carried out in two groups:

#### Group A

Locator ready-made attachment retentive insert with medium(standard/regular) retention values (control group).

#### Group B

Digitally fabricated locator custom-made attachment retentive insert.

**Fig. 7:**
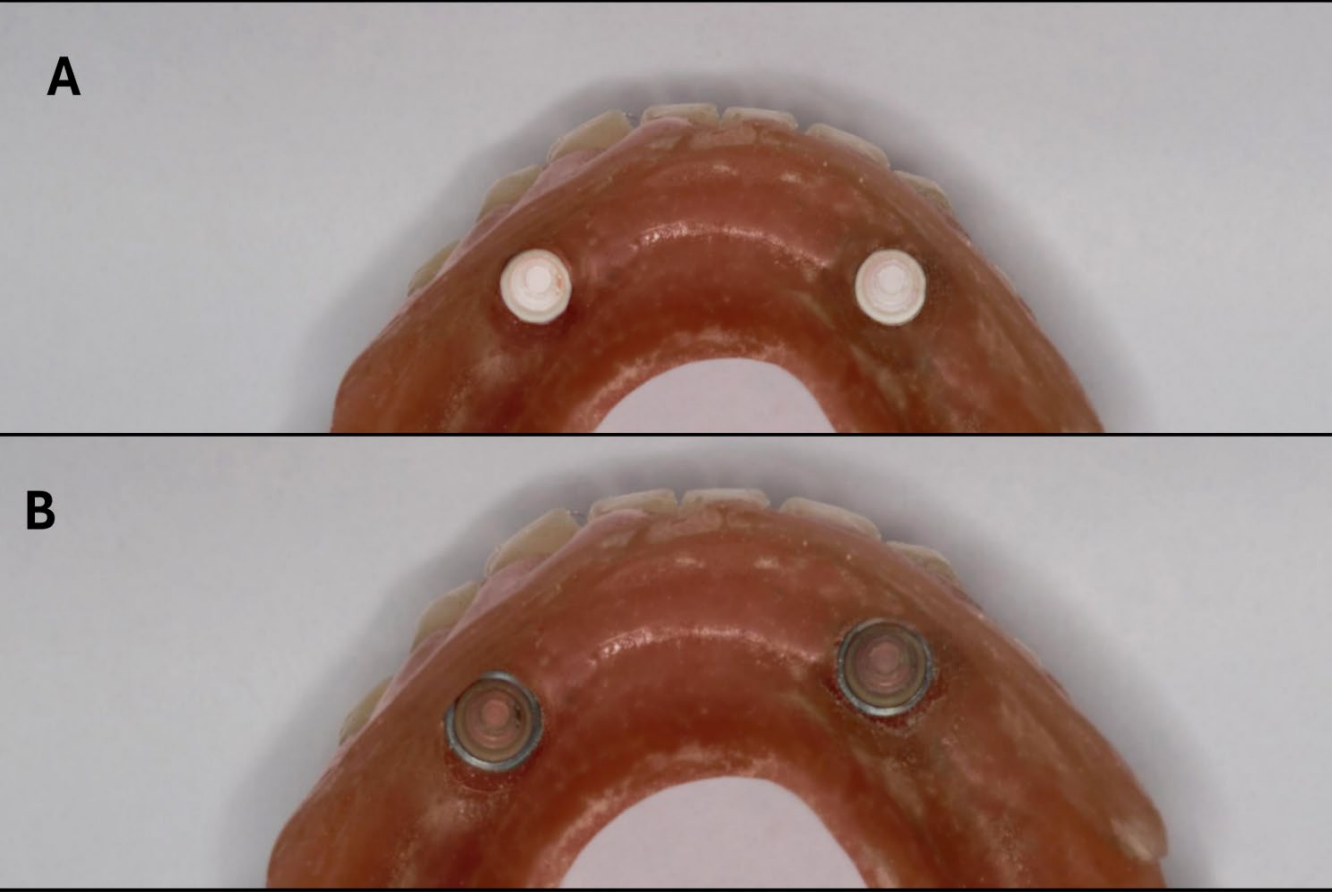
Picked up locator retentive insert. **A**: Picked up custom-made locator retentive insert (white in color) in the denture fitting surface. **B**: Picked up a ready-made locator retentive insert (clear in color) in the denture fitting surface

The pickup of the retentive inserts was performed using cold-cured acrylic resin after ensuring complete denture seating over the model (Fig. 7). The denture seating was checked through the area of the retromolar pads. For the ready-made one, the pickup procedure was performed using the metal housing and lap-processing retentive inserts, while the custom-made retentive inserts were picked up directly to the denture base without metal housing.

### Fatigue test

Each model with its denture submerged in artificial saliva was fixed to the lower fixed compartment of the universal testing machine (Model 3345; Instron Instruments Ltd., USA). The insertion and removal cycles were performed at a crosshead speed of 50 mm/min with a load cell of 500 N [5, 15]. The test repeated 720 and 1440 cycles to clinically simulate six and twelve months of insertion and removal, according to previous studies [17, 18]. 

**Fig. 8:**
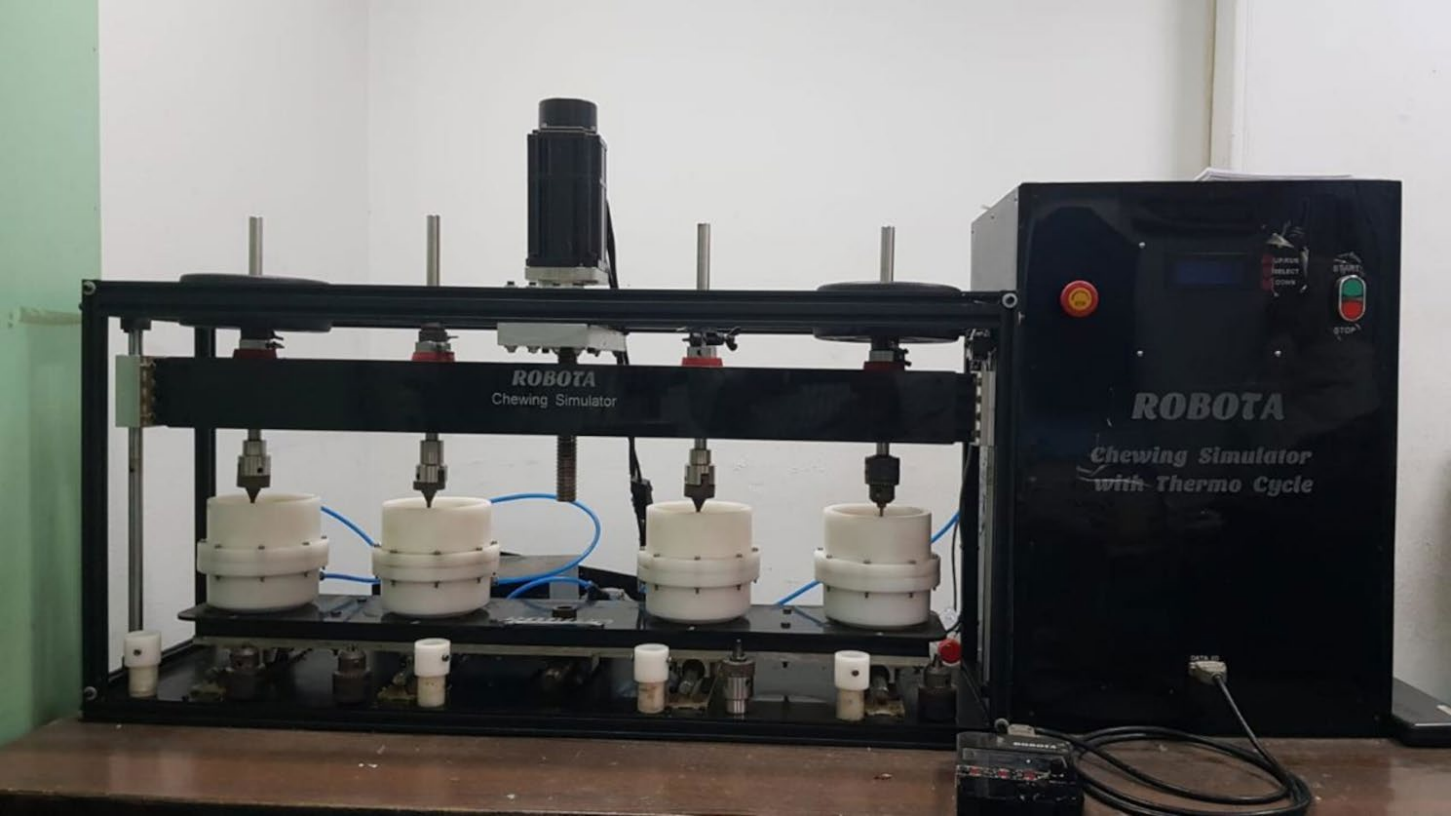
The dentures inserted in the chewing simulator

To perform the cyclic loading test, the four-station multimodal ROBOTA chewing simulator (AD-TECH TECHNOLOGY CO., GERMANY) was used to perform 120,000 and 240,000 cycles, respectively to simulate 6 months and 1 year of clinical use. The machine parameters were 0.5 and 5 mm horizontal and vertical paths respectively at a speed of 60 mm/ sec with a force of 68.6 N [19]. (Fig. 8).

The load required to dislodge the sample was recorded in Newton, and the data were recorded using computer software (Bluehill Lite; Instron Instruments) [17]. 

#### Statistical methodology

Statistical analysis was performed using SPSS 16 (Statistical Package for Scientific Studies). The normality test was performed using the Shapiro-Wilk test and the Kolmogorov-Smirnov test, which revealed a normal distribution of data. Accordingly, a comparison between the two studied groups was performed using an independent sample t-test.

## Results

**Fig. 9:**
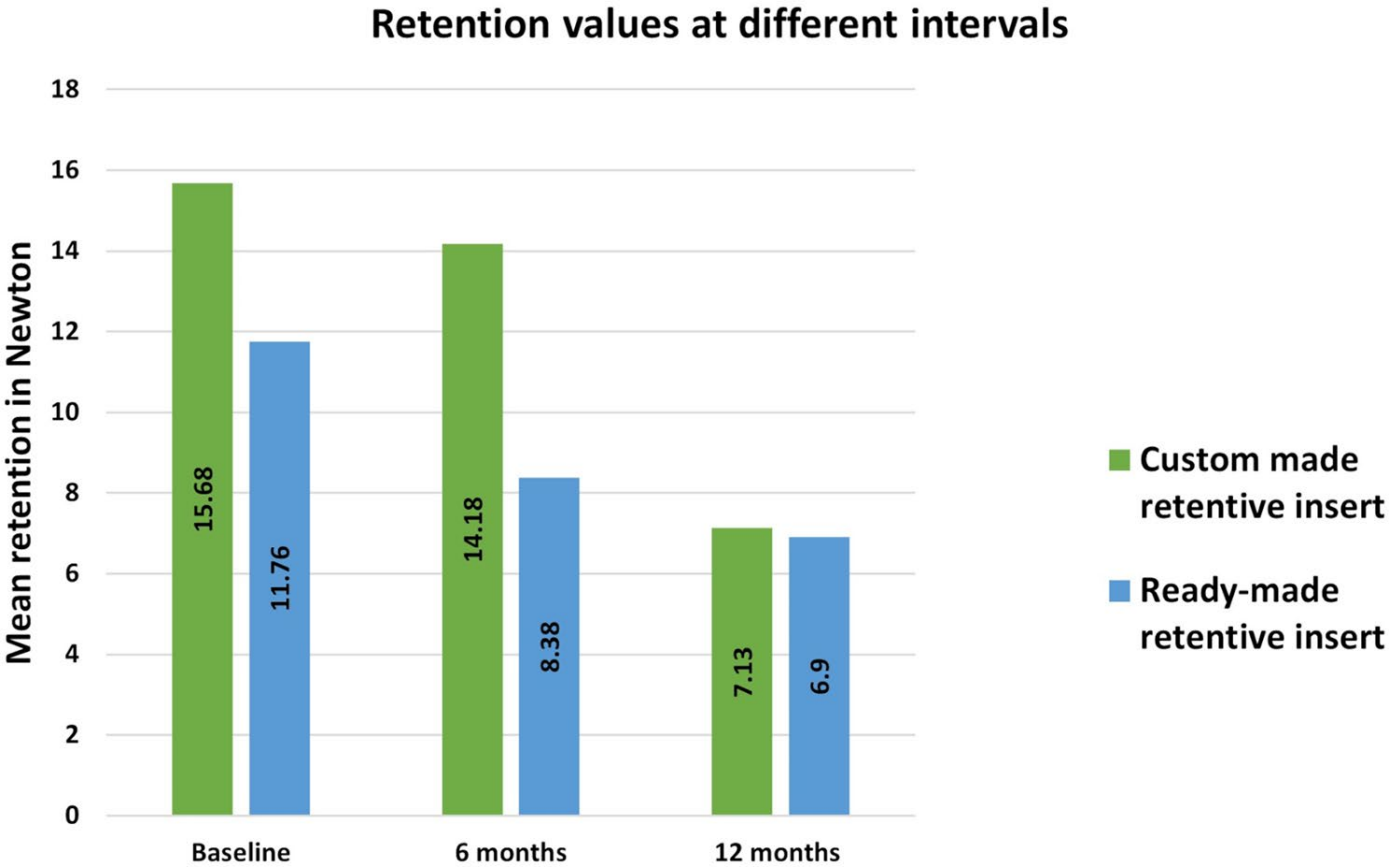
Bar chart of mean retention values in (Newton) comparing the two studied groups at different time intervals at baseline, 6 months, and 12 months of simulated clinical use

The custom-made attachment retentive insert group (15.68 ± 2.8 N) showed significantly higher retention than the ready-made group (11.76 ± 1.51 N) (*P* = 0.01). After 6 months of simulated clinical use, the custom retentive insert (14.18 ± 2.65 N) showed significantly higher retention than the ready-made one (8.38 ± 0.8 N) (*P* = 0.0001). But after 12 months, there was an insignificant difference between them as *P* = 0.83.as seen in Table 1 and (Fig. 9).

**Table 1:** Mean and standard deviation of retention at different intervals in both studied groups

		Custom-made retentive insert		Ready-made retentive insert		Mean Difference	Std. Error Difference	95% Confidence Interval of the Difference		P value
		Mean	Standard Deviation	Mean	Standard Deviation			Lower	Upper	
Retention	Baseline	15.68	2.8	11.76	1.51	3.92	1.30	1.02	6.82	0.013*
	6 months	14.18	2.65	8.38	0.8	5.80	1.13	3.29	8.32	0.0001*
	12 months	7.13	0.82	6.90	2.45	0.23	1.05	-2.12	2.58	0.833

*Significant difference as *P* < 0.05

## Discussion

This study evaluates a fully digital solution for fabricating a custom-made locator retentive insert as a replacement for a ready-made one or those no longer supplied by the manufacturer, and the authors are unaware of any previous studies on the fully digitally designed and manufactured locator attachment retentive insert.

The challenges addressed in the current study were to use the current digital technology and free-form nondental software (Meshmixer) to design a retentive insert that has a negative replica of the locator attachment geometry which provides a spacer between the retentive insert and the locator attachment to allow for some degree of mobility of the denture and act as a stress breaker. Finally, the scarcity of previous studies that discussed the digital fabrication of the locator retentive insert was very challenging while writing this report.

The use of a computer-guided implant placement stent standardizes research work by controlling the implant position in all the study models according to the prosthetically driven implant placement concept [20, 21]. Locator attachment has the ability to overcome many anatomical and surgical limitations that can affect implant angulation and position through its ability to correct implant angulations up to 20 degrees. It can also be used in limited inter-arch space, which makes it the attachment of choice in most clinical overdenture situations [22]. 

It was clear in mind when designing the locator retentive insert to use CAD-CAM technology to fabricate the retentive insert with outer undercuts to provide mechanical interlocking with the denture fitting surface in the pickup stage [14, 23]. 

It was reported by previous studies that the minimum retention of implants retained over dentures that provide patient satisfaction ranges from 5 N to 8 N [24, 25]. Therefore, studies that evaluate the selection of attachment retentive inserts are very important for the long-term success of the prosthesis with fewer maintenance visits [26]. 

The wear of the attachment retentive insert occurs as a result of continuous friction between the metal attachment abutment and the polymeric retentive insert during masticatory cycles, overdenture insertion, and removal [12]. Also, the use of artificial saliva in this study had a great role in mimicking the real clinical situation by washing the debris away and acting as a lubricant, which in turn decreased the amount of retentive insert wear [5]. Also, the retention values were measured using the universal testing machine Instron (IS), which is a reliable instrument to test retention forces as reported by many in vitro studies [27–29]. 

A recent study by Nassar HI and Abdelaziz MS [30] tested PEEK material as an attachment retentive insert and concluded that PEEK material was a candidate for bar retentive insert due to its unique mechanical properties and high wear resistance; therefore, PEEK was our material of choice to fabricate the custom locator attachment retentive insert [31]. 

It was clear in mind when selecting the ready-made locator retentive insert(control group) to use the transparent one with regular or standard retention as was reported in previous studies by Abdelaziz et al. [5] due to its favorable retention in axial and non-axial loading and its routine use in implant overdenture prostheses [32]. Last but not least and most importantly, during designing the custom-made peek retentive insert, a spacer offset of 0.03 mm was created, this spacer allowed for some degree of mobility and also controlled the retention values. Many trials were conducted to reach an optimum spacer amount that nearly the same amount in the regular ready-made retentive insert.

The present study reported a significant difference in the retention values at baseline and after 6 months of simulated clinical use, where the custom-made retentive insert showed higher retention values than the ready-made retentive insert. On the other hand, there was an insignificant difference after 1 year of simulated clinical use. This result is in accordance with several studies. Nassar HI and Abdelaziz MS [30] reported in their study, which compared custom-made bar retentive inserts with ready-made ones, that there were significant differences at the start of the study but no significant differences after 3 years of simulated use. Also, Bayer et al. [7] reported no statistically significant difference after conducting a randomized clinical trial between the PEEK and Polyoxymethylene bar retentive inserts. The insignificance difference after follow-up could be attributed to the wear behavior of the milled peek retentive inserts which may need investigations of future studies. The difference between our study and the previous studies is that those studies were conducted on bar attachment custom retentive inserts, but we conducted this study on locator attachment retentive inserts as it is the most commonly used dental attachment.

During regular maintenance visits, when the locator abutments are worn out and the retentive inserts don’t provide adequate retention or are no longer available by the manufacturer in some countries. The use of custom-made PEEK retentive inserts provides an out-of-the-box cost-effective solution. The cost to manufacture one custom-made retentive insert ranges from 10 to 15 dollars which is nearly a quarter of the price to change both the locator abutment and its retentive insert. The manufacturing of a large number of custom attachments will also decrease the cost of manufacturing. Also, the time to manufacture a custom-made attachment ranges from 2 to 3 h including scanning, designing, and manufacturing which is much less time in comparison to importing a new attachment. A varied number of custom-made attachments can be produced with varied outer designs and retentive forces at nearly the same time as producing one retentive insert.

One of the oblivious disadvantages of the introduced custom-made retentive inserts is that they are picked directly into the denture fitting surface without metal housing which makes the removal of the retentive insert difficult during follow-up visits.

However, we did not compare the custom-made locator attachment with the different color-coded (blue and pink) ready-made retentive insert and used only the clear retentive insert which may be considered as a limitation of this study. But this point was clear in mind as was reported by many studies that the different color-coding systems proposed by the manufacturer aren’t reliable enough to differentiate between different retention values as there was no statistically significant difference between d retentive inserts with different color codes [5, 33]. 

The study conducted in this report is an in vitro study that cannot mimic all the clinical situations such as oral environment, acidic food consumption by the patient, and patient parafunctional habits, which may affect the retention values of the studied attachment retentive inserts. On the other hand, this in vitro study can focus on the performance of the custom retentive insert and compare it to the ready-made one in a controlled environment. Another limitation of this study is that the fabrication of the custom-made retentive insert requires a skilled dental designer familiar with the designing software program, the CAM software settings, and the milling machines themselves. Another limitation of this study includes the follow-up of 1 year for retention values therefore it is recommended to perform in vivo clinical trials for long-term follow-up with a large sample size for generalizability of the study results, also it is advised to test the wear behavior and retentive force of the custom-made attachments with different implant angulations rather than the used zero degree angulation used in this study which is considered a limitation .

## Conclusions

According to the results of this study, it could be concluded that:

- The custom-made digitally designed and fabricated locator attachment retentive insert could be used as an alternative to the ready-made one with the implants placed parallel to each other at a zero-degree angle to the model.
- The custom-made digitally designed locator attachment retentive insert offers a prosthetic solution for overdenture maintenance when the ready-made ones are no longer available from the manufacturer.
- It is recommended to evaluate the custom-made retentive inserts in long-term clinical trials with different implant angulations.

## Data Availability

The datasets used and/or analyzed during the current study are available from the corresponding author upon reasonable request.
